# Insights from the molecular docking and simulation analysis of seocalcitol with ACE2 and vitamin D receptor

**DOI:** 10.6026/9732063002001480

**Published:** 2024-11-05

**Authors:** Sumanpreet Kaur, Deepak Yadav, Sukhpreet Singh, Monu Kumari, Deepak Kumar, Kirtimaan Syal, Rajasri Bhattacharyya, Dibyajyoti Banerjee

**Affiliations:** 1 Department of Experimental Medicine and Biotechnology, Postgraduate Institute of Medical Education and Research, Chandigarh- 160012, India; 2 Department of Biological Sciences, Birla Institute of Technology and Science-Pilani, Hyderabad campus, Hyderabad, Telangana -500078, India

**Keywords:** Angiotensin converting enzyme 2 (ACE2), Vitamin D, Seocalcitol, COVID-19, molecular docking, vitamin D receptor (VDR)

## Abstract

COVID-19 has caused a severe impact on global health. Several studies have reported the use of Vitamin D in the management of COVID-19 illness; however, the molecular basis of its action is not clear. This draws an interest to understand the molecular insights of binding of vitamin D and its analogues with ACE2 (angiotensin-converting enzyme 2) and VDR (vitamin D receptor) using molecular docking analysis. Here, we have documented the molecular docking and simulation analysis of the binding interactions of seocalcitol with ACE2 and VDR for further consideration in managing COVID-19.

## Background:

The COVID-19 disease is caused by SARS-CoV-2 (a respiratory pathogen), which has emerged as a serious health challenge around the world [1]. The virus, through its S-protein, binds to Angiotensin-converting enzyme 2 (ACE2) receptor, down regulates it, and enters the host cell [2]. The COVID-19 illness causes respiratory distress and which can lead to mortality [3]. Vitamin D deficiency has been reported in COVID-19 illness. Hence, understanding the interplay of Vitamin D status in COVID-19 disease has gained attention [4- 5]. Vitamin D inhibits renin from forming angiotensin I, and this subsequently limits the formation of angiotensin II [6]. Overall, it down regulates ACE [7] and up regulates ACE2 expression in host cells [8], making them susceptible to viral S-proteins, a contradicting paradigm related with Vitamin D supplementation. Its supplementation has not been proven to be beneficial in the context of its clinical relevance [9]. Therefore, it is of interest to report the binding of vitamin D analogues with ACE2 and VDR to design a strategy to manage COVID-19 infection.

## Methodology:

## Ligand structures:

The 3-dimensional structures of vitamin D and its analogues were downloaded from the PubChem database. The PubChem ID of the compounds is mentioned against each of them. Vitamin D-5280453; Analogues: Paricalcitol-5281104, Doxercalciferol-5281107, Falecalciferol-5282190, Maxacalcitol-6398761, Tacalcitol-5283734, Calcipotriol-5288783, Alfacalcidol-66577031, Eldecalitol-6918141, Seocalcitol-5288149, Lexacalcitol-5288670 and Inecalcitol-6915835.

## Target structures:

ACE2 (6LZG) and VDR (1DB1) structures (3-dimensional) were downloaded from the Protein Data Bank.

## Molecular docking:

Autodock (version 4.2.6) was used to perform a single docking run and it was also used to screen the binding interaction of ligands with ACE2. The screened analogues were then again docked with ACE2 and VDR using two different docking software (Autodock and GOLD (version 5.3)) for their comparison with vitamin D. For ACE2; Lys31, whereas for VDR; Tyr143 and His305 were selected as the active site for performing docking [9, 10- 11].

## Molecular Dynamics (MD) Simulation of docked complexes:

MD simulations (using GROMACS software) for 10 ns were carried out to check the stability of the docked complexes. The best docked structures from Auto-dock and GOLD docking were used for the MD run [10]. Docked complexes were visualized using Pymol. To analyze the obtained MD simulation parameters like root-mean-square deviation (RMSD), radius of gyration, hydrogen bonds, and potential energy, Xmgrace plotting tools were used.

## Results and Discussion:

Initial screening reveals that Seocalcitol, a vitamin D analogue, exhibits a more favourable binding free energy (-6.79 Kcal/mol) with ACE2 (Lys31) compared to Vitamin D (-5.77 Kcal/mol) and its other analogues. Among the compounds analyzed, the binding energies ranged from -4.94 kcal/mol for Eldecalcidol to -6.52 kcal/mol for Doxercalciferol, with Seocalcitol showing one of the most negative values. This strong binding affinity suggests that Seocalcitol interacts more robustly with ACE2, indicating its therapeutic promise. Further, the docking experiments conducted using AutoDock and GOLD software consistently demonstrated that Seocalcitol binds more effectively to ACE2 and VDR than standard Vitamin D (Table 1). Notably, the orientation and binding energy values remained consistent across both docking tools, strengthening the credibility of the results. The interaction was found to be more when the docking grid was centred on His305, further emphasizing the strong and specific binding of Seocalcitol (Table 1). In all docked complexes involving ACE2 and VDR, Seocalcitol formed at least one hydrogen bond. These were supplemented by several non-bonded interactions, likely including van der Waals forces and electrostatic attractions, which contributed to the overall stability of the complexes. This stability was further corroborated by molecular dynamics (MD) simulations. The MD simulations confirmed that the Seocalcitol-ACE2 and Seocalcitol-VDR complexes remained stable over time. A decreasing radius of gyration indicated the formation of more compact and stable complexes, while the RMSD stabilised after approximately five nanoseconds. Moreover, one hydrogen bond persisted throughout the simulation period in both complexes, reinforcing the reliability of the binding interactions observed during docking. Altogether, these findings suggest that Seocalcitol binds more strongly and maintains a stable interaction with ACE2 and VDR, outperforming other analogues in affinity and stability. This supports its potential as a superior therapeutic candidate, particularly for diseases involving these receptors, such as COVID-19 (via ACE2) and osteoporosis or other bone-related disorders (via VDR). These observations are consistent with the data presented in Table 1 and further supported by prior docking and MD studies, such as those referenced in https://www.researchsquare.com/article/rs-63402/v1. Vitamin D has been widely studied for its role in managing COVID-19, with both supporting and opposing perspectives being reported. While some evidence suggests a beneficial effect, others argue against its utility [12]. This reflects a paradoxical relationship. On one hand, Vitamin D downregulates the renin-angiotensin system (RAS), particularly by reducing renin levels and, consequently, angiotensin II-a molecule associated with inflammation and lung fibrosis. On the other hand, Vitamin D is known to upregulate ACE2 (receptor utilized by SARS-CoV-2 to enter host), and thus potentially increases susceptibility to infection. Our findings suggest that Seocalcitol may offer a strategic advantage in this paradox. Its stronger binding to VDR may enhance its renin-inhibiting effects, potentially reducing pulmonary complications such as inflammation and fibrosis associated with COVID-19 [13-14]. Furthermore, Seocalcitol is predicted to occupy the viral binding site at Lys31 on the ACE2 receptor more effectively than standard Vitamin D. This site is crucial for SARS-CoV-2 entry into host cells. By occupying this site, Seocalcitol could potentially prevent viral attachment and entry which diminishes the risk of infection, if administered before exposure. Thus, Seocalcitol may serve dual roles-preventing virus binding to ACE2 and attenuating downstream inflammation through RAS modulation. These combined effects highlight its therapeutic promise and the urgent need for experimental validation of these computational predictions.

## Conclusion:

Data show that seocalcitol (a vitamin D analogue) exhibits optimal binding features with ACE2 and VDR.

## Figures and Tables

**Table 1:** Binding energy (Kcal/mol) and GOLD score of Vitamin D and Seocalcitol docked at Lys31 of ACE2 and Tyr143 and His305 of VDR with AutoDock and GOLD. ***p value<0.0001

**Software**	**Ligand**	**ACE2**	**VDR**	
		**Lys31**	**Tyr143**	**His305**
AUTODOCK	Vitamin D	-5.1 ± 0.60 (n=60)	-7.85 ± 0.67 (n=60)	-9.89 ± 0.46 (n=60)
	Seocalcitol	-5.81 ± 0.67 (n=60)***	-6.81 ± 0.81 (n=60)	-10.49 ± 0.67 (n=60)***
GOLD	Vitamin D	34.18 ± 6.79 (n=29)	44.18 ± 8.19 (n=50)	47.25 ± 4.58 (n=50)
	Seocalcitol	39.65 ± 7.91 (n=31)***	52.52 ± 5.83 (n=39)***	53.09 ± 4.53 (n=47)***
